# Representation of the Numerosity ‘zero’ in the Parietal Cortex of the Monkey

**DOI:** 10.1038/srep10059

**Published:** 2015-05-22

**Authors:** Sumito Okuyama, Toshinobu Kuki, Hajime Mushiake

**Affiliations:** 1 Department of physiology, Tohoku University School of Medicine, Sendai 980-8575, Japan; 2 Department of neurosurgery, Southern Tohoku General Hospital, Miyagi 989-2483, Japan

## Abstract

Zero is a fundamental concept in mathematics and modern science. Empty sets are considered a precursor of the concept of numerosity zero and a part of numerical continuum. How is numerosity zero (the absence of visual items) represented in the primate cortex? To address this question, we trained monkeys to perform numerical operations including numerosity zero. Here we show a group of neurons in the posterior parietal cortex of the monkey activated in response to numerosity ‘zero’. ‘Zero’ neurons are classified into exclusive and continuous types; the exclusive type discretely encodes numerical absence and the continuous type encodes numerical absence as a part of a numerical continuum. “Numerosity-zero” neurons enhance behavioral discrimination of not only zero numerosity but also non-zero numerosities. Representation of numerosity zero in the parietal cortex may be a precursor of non-verbal concept of zero in primates.

Mathematical history suggests that before the development of the notation system of zero, humans seemed to recognize the concept of zero without a symbol^1^,^2^,^3^. Even today, one Amazonian indigenous group with a limited notation system for numbers and no word for zero can understand empty sets by the expression: “there is nothing left”^4^. Four-year-old children prior to understanding symbolic zero can order numerosities including empty sets^5^. Moreover, non-human primate research shows monkeys can distinguish empty objects from existing objects^6^,^7^. Therefore, the concept of zero seems to exist with other non-zero numerosities without the notation system in animal and humans alike^8^,^9^,^10^,^11^,^12^.

How is the concept of zero represented in the brain? Non-zero numerical representations were revealed from monkeys’ parietal cortex and frontal cortex in single neuron recording studies^13^,^14^,^15^,^16^,^17^,^18^,^19^. Computational models have suggested that non-zero numerosities were represented in either labeled-line coding or monotonic coding^20^,^21^,^22^. Numerosity zero could be coded parametrically as an extension of non-zero numerosities on the same numerical continuum (i.e., 0, 1, 2, 3, …). However zero is special among numerosities, because zero is not a counting number and because zero represents the absence of quantity^23^. It is also possible that numerosity zero could be coded as binary^24^ (i.e., absence or presence). In a behavioural study, a chimpanzee was able to use the numerical zero symbol in both an “absence or presence” scheme in a cardinal context and in a numerical continuum scheme in an ordinal context. However, transfer between these two meanings of zero has been found to be incomplete in use of the zero symbol^25^. This study suggests that two different concepts of zero might also be context-specific in non-human primates. Therefore, it is still a big question whether zero is represented explicitly in the brain. To investigate this issue, we devised a numerical operation task including numerosity zero. Numerosity zero is special in number and would be encoded in a numerosity selective manner. Thus we explored and analyzed cellular activity in the ventral intraparietal area (VIP), in which numerosity-selective encoding is demonstrated in single neuron recording studies for numerical cognition^16^,^18^,^19^.

## Results

**Figure 1:**
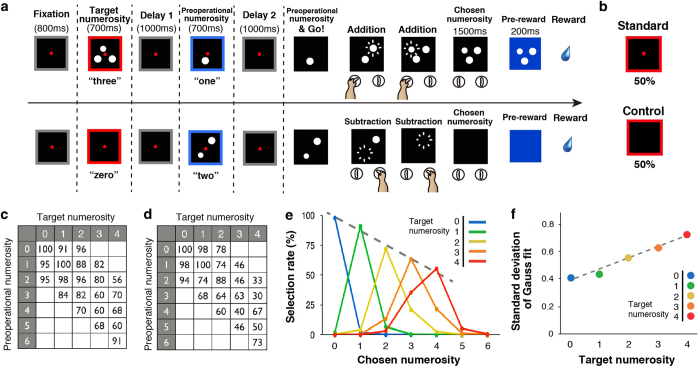
Schematic illustration of the numerical operation task and behavioral performance. (**A**) This task began when the monkey focused on a fixation point. The presentation of a red square containing circles represents the target numerosity. After Delay 1, a blue square containing the second numerosity was displayed as an preoperational numerosity. After Delay 2, the monkeys were required to remember the target numerosity and manipulate the device for the purpose of matching the preoperational numerosity with the target numerosity. Monkeys were allowed to manipulate either the right or left device, one at a time, as many times as they chose. The number of circles displayed after 1,500 ms of inactivity was defined as the chosen numerosity. If the chosen numerosity matched the target numerosity, a reward was delivered. The relationship between using the right/left device and adding/subtracting the numerosity was determined by either of the two rules (see Fig. S1B for details). (**B**) Two types of stimuli for target numerosity of zero were randomly used. (**C** and **D**) Twenty-two numerosity pairs were prepared during recordings. The number inside the box indicates overall percent correct trials for that pair [for both monkeys (**C**) and (**D**)]. **(E)** Average selection rate of both monkeys are shown according to the target numerosity (Chance = 20%). The dashed line represents the best-fit linear model. (**F**) The standard deviation of the Gaussian fitting plotted against the target numerosity. The dashed line represents the best-fit linear model.

We trained two monkeys to perform a numerical operation task^26^ (Fig. 1A,B and Fig. S1 Supplemental Information) (see Methods). Monkeys were required to remember the first quantity (Target numerosity) and manipulate the second quantity (Preoperational numerosity) for the purpose of exactly matching two quantities. The target numerosity and the preoperational numerosity were 0–4 and 0–6, respectively (Fig. 1C,D) followed by delay periods. After a Go signal, monkeys were allowed to increment or decrement the number of objects by manipulating either the right or left device, one at a time, as many times as they chose. After operating either device, if they halted the device and fixated on the screen for 1.5 s while waiting for a visual feedback of match/non-match, the displayed numerosity was taken as the monkeys’ chosen numerosity. If the chosen numerosity matched the target numerosity, a reward was delivered. The visual numerical objects used consisted of white circles on a black background, appearing in random locations among the possible positions in every trial. Numerosity zero was presented as empty sets in a black background on the screen. To exclude the possibility that the monkeys used non-numerical information to perform the matching task by attending to low-level visual features (rather than to numerosity), we used one standard signal and one control signal (stimuli of different format, either of same circumference or all aligned) alternatively^15^,^16^. The exact physical appearance of each numerical quantity was counterbalanced across these sets with regard to area, circumference, density, and configuration (same circumference stimulus: decreased area and equal circumference as a function of numerosity, linear stimulus: control for density and configuration) (Fig. S1A Supplemental Information). Standard and control signals appeared in random order at random positions on the screen. For numerosity zero, there was a possibility that monkeys counted the fixation point as one, therefore we prepared numerosity zero without the fixation point. Two types of signals for numerosity zero (standard and control) were used randomly (Fig. 1B). Both monkeys performed better than chance for all tested target numerosities (Fig. 1E, Binomial test, P < 0.01). The monkeys’ success rates linearly declined (linear regression, *r*^2^ = 0.97, *P* < 0.01) and chosen numerosity became more variable as a function of the target numerosity including numerosity zero. There is a statistically significant linear relationship between the target numerosity and the standard deviation of chosen numerosity (Fig. 1F, linear regression, *r*^2^ = 0.98, *P* < 0.01), suggesting the numerical size effect. We also compared tuning of chosen numerosity between standard versus control trials and whether tuning was different in the two trial types. We found a decrease in success rates as a function of the target numerosity for both signal types (Fig. S2 A&B Supplemental Information), showing similar tuning features. There was not a statistical significant difference in performance for standard signal and control signal (72.0% vs 72.6%, respectively; Chi-square test, P = 0.21). This suggests that monkeys were incorporating empty sets into the number line.

**Figure 2:**
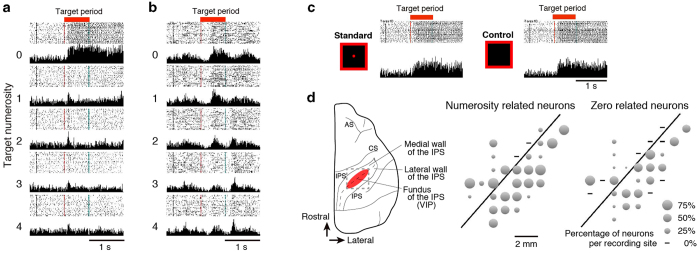
Activity of VIP cells selective for numerosity zero during the target period. Raster displays and peri-event histograms illustrating the cellular activity for numerosity 0–4 during the target period (red square). (**A**) Exclusive activity to numerosity zero. (**B**) Continuous activity through numerosity zero to four. (**C**) Responses of the neuron shown in (**A**) to the stimuli with or without the fixation point. The activity of this neuron showed no significant difference between two types of zero stimuli. (**D**) Left: the dorsal view of the hemisphere shows the extent of the surveyed area in the exposed intraparietal sulcus. Recording sites of numerosity related neurons (middle) and zero related neurons (right).

We recorded 614 cells in the VIP of a monkey performing a numerical operation task. In the VIP, cellular activity selective to the target numerosity 0–4 was found in 185 neurons during the target period, 101 neurons during the Delay 1 period (two-way analysis of variance (ANOVA), two factors: target numerosity × stimulus property, the only main effect of target numerosity, *P* < 0.01, see Methods). Among these, 137 neurons showed differential activities to numerosity zero (‘zero’ neuron) (target period, 99/185; Delay 1 period, 76/101, see Methods). We found two types of ‘zero’ neurons in the VIP exhibit changes in activity during the presentation of the target numerosity of zero (Fig. 2). The first type of ‘zero’ neurons exclusively represented empty sets or numerosity zero. An example of the first type of ‘zero’ neurons showed a rapid buildup in activity and sustained through Delay 1 period in response to numerosity zero (Fig. 2A). In contrast, the same neuron was consistently silent to other numerosities of 1 to 4. The sustained activity for numerosity zero and non-zero is binary and we call this an exclusive type. The second type of ‘zero’ neurons encodes numerosity zero and non-zero numerosity continuously. An example of the second type of cells showed peaked activity to the numerosity zero and gradually decreased its activity to other numerosities (Fig. 2B). We call the second type of neurons a continuous type. However, a question arises whether ‘zero’ neuron counted the fixation point as numerosity one? Few of the ‘zero’ neurons showed differential activities to the presence or absence of the fixation point (target period, 1/99; Delay 1 period, 1/76; one-way ANOVA, *P* < 0.01, see Methods) (Fig. 2C), therefore we confirmed that neuronal activities were determined only by the absence or presence of the target numerosities.

**Figure 3:**
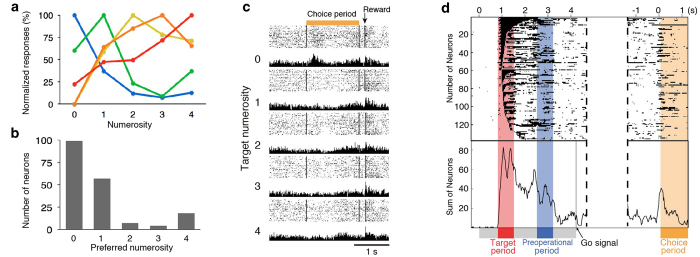
Activity of cell populations and an example of VIP cells selective for numerosity zero during the choice period (**A**) Normalized responses averaged for each numerosity related neurons for the target period. Colors are as in Fig. 1C, (**B**) Number of each preferred numerosity neurons. (**C**) Activity that increases for numerosity zero during the presentation of chosen numerosity (orange square). (**D**) Temporal patterns of target numerosity zero selectivity (as determined by a sliding two-way ANOVA) as 137 individual zero related neurons (top) and number of zero related neurons (bottom). Left: aligned by the onset of fixation period. Right: aligned by the onset of chosen numerosity.

Normalized responses of numerosity related neurons showed peaked activity at preferred numerosity and systematic decreased activity as a function of numerical distance from preferred numerosity according to Weber’s law (Fig. 3A). Therefore, ‘zero’ neurons were incorporated into the number line including ‘non-zero’ neurons. The number of ‘zero’ neuron was the most dominant and followed by that of numerosity ‘one’ neurons (Fig. 3B).

Interestingly, during the choice period, we found ‘zero’ neuron encoding target numerosity zero again (Fig. 3C). To explore the time course of target selectivity of ‘zero’ neurons, we calculated target zero selectivity in sliding windows of 100 ms moving in steps of 25 ms (Fig. 3D) (see Methods). After the presentation of target zero, the sum of target zero selective neurons increased before and during the preoperational period (e.g., Fig. S3A Supplemental Information). One third of ‘zero’ neurons (54 of 137 zero related neurons) were reactivated when the monkey chose numerosity zero during the choice period. The onset of increase in number of target zero selective neurons during the choice period preceded the appearance of numerosity zero on the screen (Fig. 3D). Therefore the selectivity seems to reflect the prediction of a numerical outcome. Moreover, ‘zero’ neurons also encode the preoperational numerosity of zero during the preoperational period (Fig. S3 B&C Supplemental Information) (see Methods). Overall, these results support that VIP neurons represent numerosity zero as well as non-zero numerosity.

**Figure 4:**
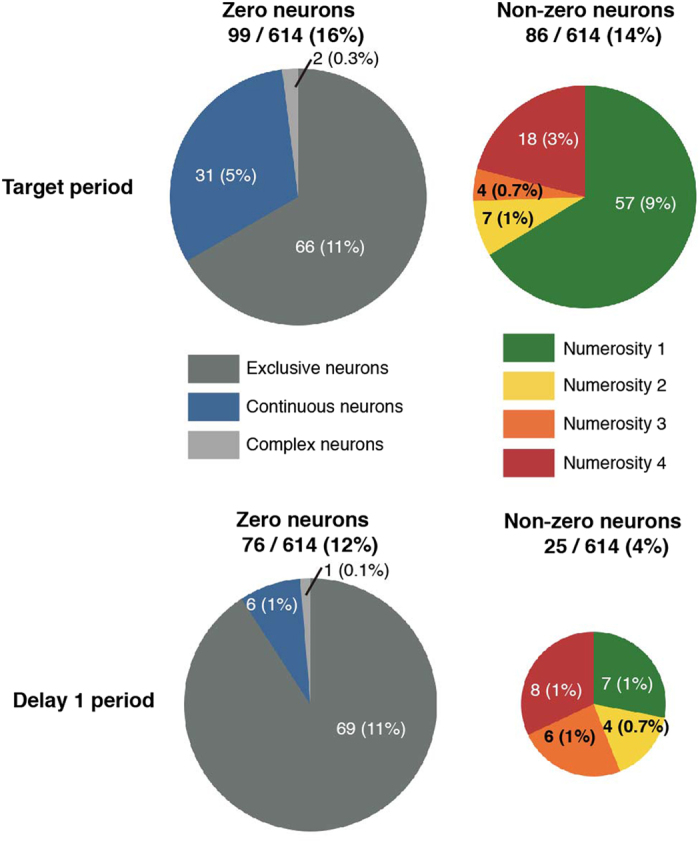
Distribution of numerosity related neurons during the target period and the delay 1 period. Pie charts illustrating the proportion of subgroups in ‘zero’ neurons (left) and ‘non-zero’ neurons (right). Classified based on the target period (top) and the Delay 1 period (bottom).

**Figure 5:**
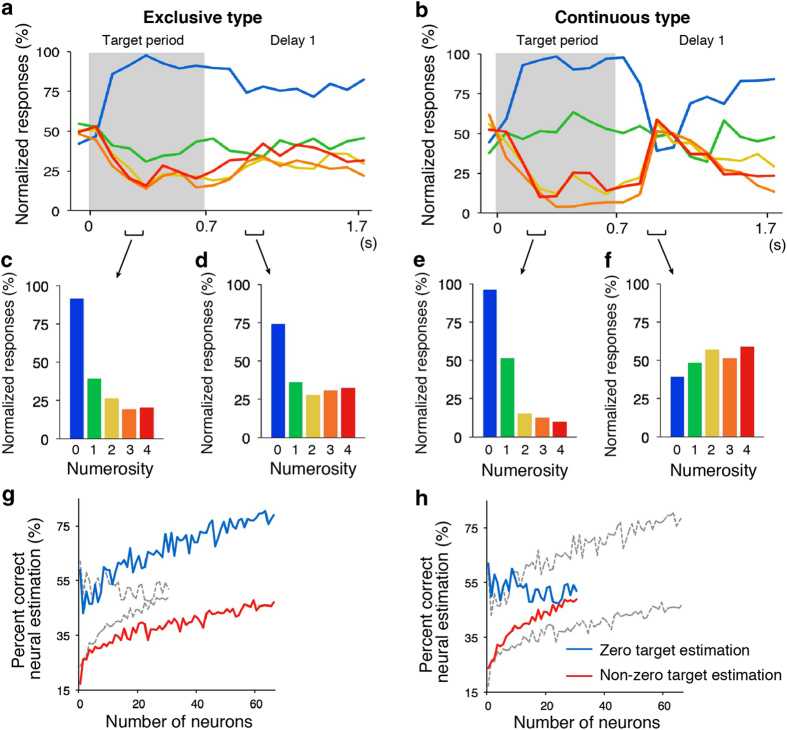
Time courses of normalized responses of two types of zero coding cell populations. (**A, C** and **D**: exclusive type, **B**, **E** and **F**: continuous type. Colors are as in Fig. 1C) (**A** and **B**) Normalized responses are shown as a function of time (sliding windows of 100 ms moving in steps of 100 ms). Gray shades represent the target period. (**C** and **E**) Normalized responses during 200–300 ms after the presentation of the target numerosity are shown. Continuous type encodes numerosity one as well as numerosity zero. (**D** and **F**) Normalized responses during 200–300 ms after the disappearance of the target numerosity are shown. Continuous type shows decreased activity to numerosity zero, which is a reciprocal response from the exclusive type. Discrimination power of the exclusive type (**G**) and the continuous type (**H**) were computed with neuron-dropping analysis. The dashed curves are for comparison of each figure. The exclusive type shows a steeper slope than the continuous type in the estimation of numerosity zero trials (blue), however shows a lower gradient in the estimation of non-zero numerosity trials (red).

To make further analysis for the numerical selectivity of ‘zero’ neurons, we classified ‘zero’ neurons into either an exclusive or continuous type based on the response to the target numerosities 1 to 4 (see Methods) (Fig. 4). Two-thirds of ‘zero’ neurons (66/99) were classified as a discrete type. One-third of zero coding neurons (31/99) were classified as a continuous type. We first calculated the time course of normalized response for each target numerosity (Fig. 5A,B). For exclusive type of ‘zero’ neuron, activity for each target numerosity was maintained during the target period and Delay 1 period. In contrast, continuous type of ‘zero’ neuron activity of each target numerosity showed transient reversal numerical preference after the disappearance of the target numerosities. To investigate numerosity preference of each type of ‘zero’ neurons quantitatively, we plotted normalized responses for each target numerosity during the early target period and during the early Delay 1 period (Fig. 5C–F). For continuous type of ‘zero’ neurons, preference of numerosity was reversed transiently during Delay 1 period (Fig. 5H: the feature is also seen in the cell of Fig. 2B). The activity may be the counter effect of adaptation to numerosity^27^,^28^.

Finally, we explored to what extent two types of zero encoding neurons contribute to estimation of target numerosity in the population level, we conducted a neuron-dropping analysis^29^,^30^for zero and non-zero target numerosity. Neuron-dropping analysis calculated the percent correct neural estimation as a function of ensemble size. Trials of numerosity zero were estimated by discrete and continuous coding neurons. Results showed that discrete coding neurons estimated numerosity zero better than continuous coding neurons (Fig. 5G). Subsequently, trials of non-zero numerosities were estimated by two types of neurons. In contrast, the continuous type discriminated numerosities of 1–4 better than the exclusive type (Fig. 5H).

## Discussion

We found that a large proportion of VIP neurons encode empty sets or numerosity zero during the target period while the monkey was engaged in numerical operation tasks. A majority of these neurons were reactivated again to encode numerosity zero during the presentation of preoperational numerosities and during the choice period. ‘Zero’ neurons were divided into the exclusive type and the continuous type by its coding manner. The exclusive type of ‘zero’ neurons showed increased activity selectively to numerosity zero and responded only weakly to other numerosity from 1 to 4 regardless of numerical size. The continuous type of ‘zero’ neurons showed peaked activity to numerosity zero and a gradual decrease in activity according to numerical distance from zero. Population analysis showed that the activity of exclusive type of ‘zero’ neurons efficiently estimated numerosity zero better than that of the continuous type of ‘zero’ neurons, however the continuous type estimated non-zero numerosities better than the exclusive type. Therefore, ‘zero’ neurons contribute to discrimination of non-zero numerosities in addition to discrimination of zero numerosity. This study indicates that the VIP is involved in processing visually presented empty sets or ‘zero’ in addition to non-zero numerosity in numerical operation tasks.

‘Zero’ neurons may have appeared *de novo* in our study because the numerical operation task in this study required monkeys to manipulate both non-zero and zero numerosities as numerical objects. Previous reports without explicit usage of zero numerosity have shown that 17% of cells in the VIP of monkeys are selectively activated on the basis of the numerosities 1–5^16^. In our study, 14% of cells in the VIP showed selectivity to numerosities 1–4. Continuous coding neurons (5% of cells in the VIP) also code numerosity 1, therefore in total, 19% of cells are selective to numerosities 1–4. The percentage of non-zero neurons in the VIP is compatible with a percentage of numerosity-selective neurons in previous studies. Exclusive type of ‘zero’ neurons (11% of cells in the VIP) represent only numerosity zero explicitly, therefore these neurons may not be found in behavioral paradigms without the necessity of explicitly handling zero numerosity.

Continuous type of zero coding neurons showed a unique feature, a reversible response to higher numerosities such as four after the disappearance of the target numerosities. One possibility for this reversal response is that the continuous type dynamically encodes both zero numerosity and larger numerosities. However the possibility of dynamic representation seems to be unlikely, because reversal responses were only observed transiently and there were no sustained activities following this transient response, therefore the response selectivity for zero seems to be mostly maintained. Another possibility is that the continuous type of ‘zero’ neurons showed rebound effects of numerical disappearance and response preferences appeared to be temporarily reversed according to numerical distance from their preferred numerosity. This interpretation is consistent with a previous report on the effect of numerical adaptation^27^. According to that study, after adaptation to the large number of visually presented dots such as 400 dots on the screen, subjects judged that the numerosity for 100 dots was mistakenly matched with that for 30 dots. This bias of that subjective judgment of numerosity was interpreted to be due to a shifted balance between large-numerosity preferred neurons and small-numerosity preferred neurons after adaptation. It is also known that the priming of numbers or exposure to certain numerosities affect subjective judgment of quantities immediately after such exposer to numerosities in behavioral economics^31^. This is called an anchoring effect and may be due to adjustment of numerical representation to pre-exposed numerosities. Adaptation and adjustment of pre-exposed numerical representations may be two sides of a coin of context-dependent neural processing of numerosity. Further studies should be addressed about this issue.

So far we classified zero neurons into exclusive type and continuous type, according to the differentiation of response for numerosity 1 to 4. However there are alternative encoding models in numeorsity. Numerosity-selective coding assumes that individual neurons are tuned to individual numbers. Alternatively summation coding assumes that individual neurons fire monotonically stronger or weaker to increasing number^32^. According to summation coding, exclusive and continuous neurons are on a continuum with high and low slopes for exclusive and continuous neurons, respectively. This account is also consistent with our results. Additional attributes such as transient rebound activity and contribution to perception of non-zero numerosity, however, seems different in exclusive and continuous ‘zero’ neurons. These differences in two types of ‘zero’ neurons may result from number-relevant training. The validity of the interpretation should be explored in a future.

We found a comparable amount of ‘zero’ neurons and non-zero neurons in the VIP area. Specifically, 14% of VIP cells (numerosities 1–4 related neurons) were selective to numerical presence, 11% of VIP cells (exclusive coding neurons) were selective to numerical absence and 5% of VIP cells (continuous coding neurons) were selective to both numerical absence and presence. The continuous type of coding ‘zero’ neurons may contribute to the allocation of numerosity zero in a mental number line by bridging between numerical absence and presence. Thus these neurons may be the key for the mental number line including the numerosity of zero. On the other hand, the exclusive type of coding of ‘zero’ neurons contributes to explicit representation of empty sets and holds this numerical information as working memory although there were no objects to be remembered on the screen. Current results show that the visually presented empty set is represented in the VIP in two forms: analog and digital. The representations could be a precursor of non-verbal concept of zero common in human and non-human primates.

## Methods

### Behavioral task and Stimuli

We trained two monkeys (Macaca fuscata) to use one of two devices to either add or subtract the number of displayed objects in pursuit of the behavioral goal of numerical matching (see Fig. 1A). All animal care and research procedures were carried out in accordance with the Guiding Principles for the Care and Use of Laboratory Animals of the US National Institutes of Health and were approved by the Institutional Animal Care and Use Committee of Tohoku University. The goal of this task was to exactly match the numerosity of the currently displayed visual objects with the numerosity of the target objects displayed on the screen at the outset of each trial, starting from the numerosity of objects given as the preoperational numerosity. For that purpose, the monkeys operated the devices to increase or decrease the number of circles in a stepwise manner. Initially, when the monkey gazed at a fixation point on the screen for 800 ms (fixation period), 0–4 white circles appeared in a red square frame for 700 ms. This constituted the target numerosity. After a delay of 1000 ms (Delay 1 period), during which a gray square frame was shown, 0–6 circles appeared as the preoperational numerosity in a blue square frame. After the second delay of 1000 ms (Delay 2 period), during which a gray square frame with a black screen was shown again, the preoperational numerosity appeared with a tone signal. The signal triggered monkeys to either add or subtract the preoperational numerosity to match the target numerosity. Monkeys were required to gaze at a fixation point (red circle, 1.4° in visual angle) from the fixation point until the Go signal. Twenty-two combinations between the target numerosities and the preoperational numerosities were prepared and randomly used (Fig. 1C,D Supplemental Information). A clockwise rotation of the left manipulandum (left device) or a counter-clockwise rotation of the right manipulandum (right device) by the monkeys caused an increase or decrease in the number of visual objects displayed on the monitor screen. Under Rule 1, use of the left device increased the numerosity by one, whereas use of the right device decreased the numerosity by one (Fig. S1B Supplemental Information). Under Rule 2, the effects of device use were reversed and the rule switched every 230 trials. The monkeys had to repeat the operation until matching was achieved. After operating either device, if they halted the device and fixated on the screen for 1.5 s while waiting for a visual feedback of match/nonmatch, the displayed numerosity was taken as the monkeys’ decision. When the numerosity determined by the monkeys’ decision matched the target numerosity, matching was correct and a blue square appeared on the screen, followed by a juice reward. There was no time-out for the monkeys’ final decision in each trial. Monkeys could select 0–6 for the numerosity as the final decision. If the preoperational numerosity was equal to the target numerosity already appearing, the monkeys had to hold the preoperational numerosity for 1.5 s. Incorrect matching or the use of two devices at the same time led to the appearance of a red square on the screen (error signal), and a return to the beginning of a new trial. Inter-trial interval was set at 3 s, irrespective of correct or incorrect matching.

Target numerosities and preoperational numerosities are displayed in 6° × 6° red and blue square frames, respectively. Gray square frames display the delay periods. All stimuli plotted in 36 random possible locations on a 6 × 6 grid. To exclude the possibility that neurons responded to low-level visual features rather than numerosity, we used three sets of signals (standard, same circumference and linear properties, Fig. S1A Supplemental Information). Sizes of dots were randomly selected from three sizes for standard stimuli. For same circumference property, accumulation of the perimeter of all dots was constant and total area decreased as a function of numerosity. For linear properties, dots in a constant size were plotted on a line. In this property, all dots were arrayed next to each other and also used for density control.

### Recording methods

We used conventional electrophysiological techniques to obtain in vivo single-cell recordings from the VIP in the right hemisphere. Cortical sulci and recording locations were identified using a magnetic resonance imaging scanner before recording, and were verified by histological examination of Klüver-Barrera-stained brain sections. We also monitored eye positions with an infrared corneal reflection monitoring system. Neural activity was recorded using a multitrode (Thomas Recording, Giessen, Germany) and a Plexon (Dallas, TX) multi-channel acquisition processor.

### Data analysis

Our database includes cells from which activity was recorded during at least 23 correct trials for each of the target numerosities. To define numerosity related neurons, we tested the activity of each cell during the target period and Delay 1 period with a two-way analysis of variance (ANOVA, with factors: [target numerosity 0 to 4] × [stimulus type], *P* < 0.01). We defined two task periods for the numerosity related neurons: the target period (700 ms interval starting 100 ms after target numerosity presentation); and the Delay 1 period (1000 ms interval following the delay onset). Cells that varied their activities according to numerosities, irrespective of stimulus type were defined as numerosity related neurons. For cells satisfying this criterion and showed peak activity to numerosity 0, it is possible that the activity was merely a response to the black screen and we performed the second round analysis. We examined whether the cellular activity during a target period and a Delay 1 period varied from the activity during a fixation period (Wilcoxon rank-sum test, p < 0.01). For cells satisfying additional criterion, we identified numerosity zero related neurons (‘zero’ neurons) (Fig. 4).

‘Zero’ neurons were classified into exclusive type and continuous type. Again we calculated two-way ANOVA with exceptions of trials for the target numerosity zero (ANOVA, with factors: [target numerosity 1 to 4] × [stimuli], *P* < 0.01). A cell that showed no main effect of target numerosity was classified as exclusive zero. A cell that showed only main effect of target numerosity and its maximum activity was numerosity one, it was classified as continuous type (again see Fig. 4). The exclusive type shows peak activity to numerosity zero and is indifferent to numerosity 1–4. The continuous type codes numerosity zero and one continuously.

To examine the effect of a fixation point in recognition of numerosity zero, we examined the difference of cellular activities between the two types of stimuli (standard vs. control, Fig. 2C) for numerosity zero (one-way ANOVA, *P* < 0.01).

### Sliding ANOVA for ‘zero’ neurons

We performed sliding ANOVA to examine the temporal dynamics of representations in ‘zero’ neurons. Sliding two-way ANOVAs ([target numerosity 0 to 4] × [stimulus type], *P* < 0.01) for numerosity zero selectivity were calculated based on sliding windows of 100 ms moving in steps of 25 ms. Neurons that showed selective activities only to the target numerosity with maximum activity to numerosity zero was plotted as target numerosity zero related (Fig. 2B).

Moreover, to analyze selectivity to the preoperational numerosity, we included the factor of the preoperational numerosity for analysis. Sliding three-way ANOVAs ([target numerosity zero vs non-zero] × [preoperational numerosity zero vs. non-zero] × [stimulus type], *P* < 0.01) for numerosity zero selectivity were calculated based on sliding windows of 100 ms moving in steps of 25 ms. Neurons that showed selective activity for either the target zero or the preoperational zero, (or both without any effect of stimulus type) were plotted as zero related (Fig. S3C).

### Neuron-dropping analysis

We evaluated how well the target period activity of neurons discriminated the numerosity zero and non-zero numerosities. On an assumption that the monkey’s decisions were determined by the activity of the neuronal ensemble, we conducted a neuron-dropping analysis to estimate the discrimination power of VIP neurons as a function of ensemble size using the methods described previously^29^,^30^. We applied the analysis to construct the neuron-dropping curves for each of the two conditions during the target period.

## Author Contributions

S.O. designed experiments, constructed the apparatus, developed the computer programs, analyzed the data and wrote the paper. S.O. and T.K. conducted the experiments. H.M. provided ideas and designed experiments, wrote the paper and supervised the project.

## Additional Information

**How to cite this article**: Okuyama, S. *et al*. Representation of the Numerosity 'zero' in the Parietal Cortex of the Monkey. *Sci. Rep.* **5**, 10059; doi: 10.1038/srep10059 (2015).

## Supplementary Material

Supplementary Information
