# Supplementary material for: Representation of the Numerosity ‘zero’ in the Parietal Cortex of the Monkey
Source: Sci Rep. 2015 May 22;5:10059. doi: 10.1038/srep10059 (PMC4437293; doi:10.1038/srep10059)
Supplement: Supplementary Information [file srep10059-s1.pdf]

Title: Representation of the Numerosity "zero" in the Parietal Cortex of the Monkey

Authors: Sumito. Okuyama[1, 2], Toshinobu. Kuki[1] & Hajime. Mushiake[1]*

Supplementary Figures:

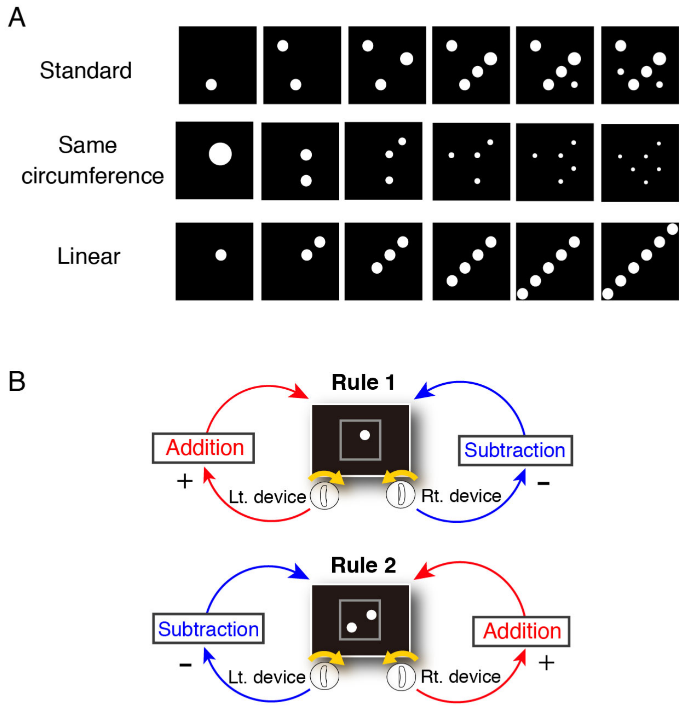

Figure S1 I Examples of three versions of stimuli (A) and schematic illustration of device use rules (B). (A)Stimuli used in this study (standard display and versions of displays with same circumference and linear properties). (B) Two rules governed the effects of device use on changes in the quantity of visual objects on the screen. Under one rule (Rule 1), a clockwise turn of the left manipulandum resulted in addition, whereas a counter clockwise turn of the right manipulandum led to subtraction. According to the second rule (Rule 2), the effect of using each device was reversed.

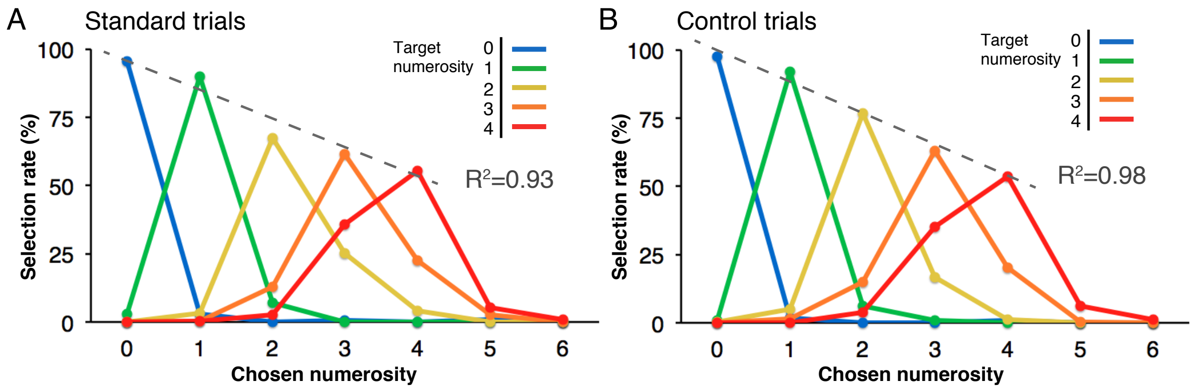

Figure S2 I Behavioral performance curves for the standard signal (A) and the control signal (B). Average selection rate of both monkeys are shown according to the target numerosity. The dashed line represents the best-fit linear model (linear regression, A: r^2 = 0.93, P < 0.01, B: r^2 = 0.98, P < 0.01)

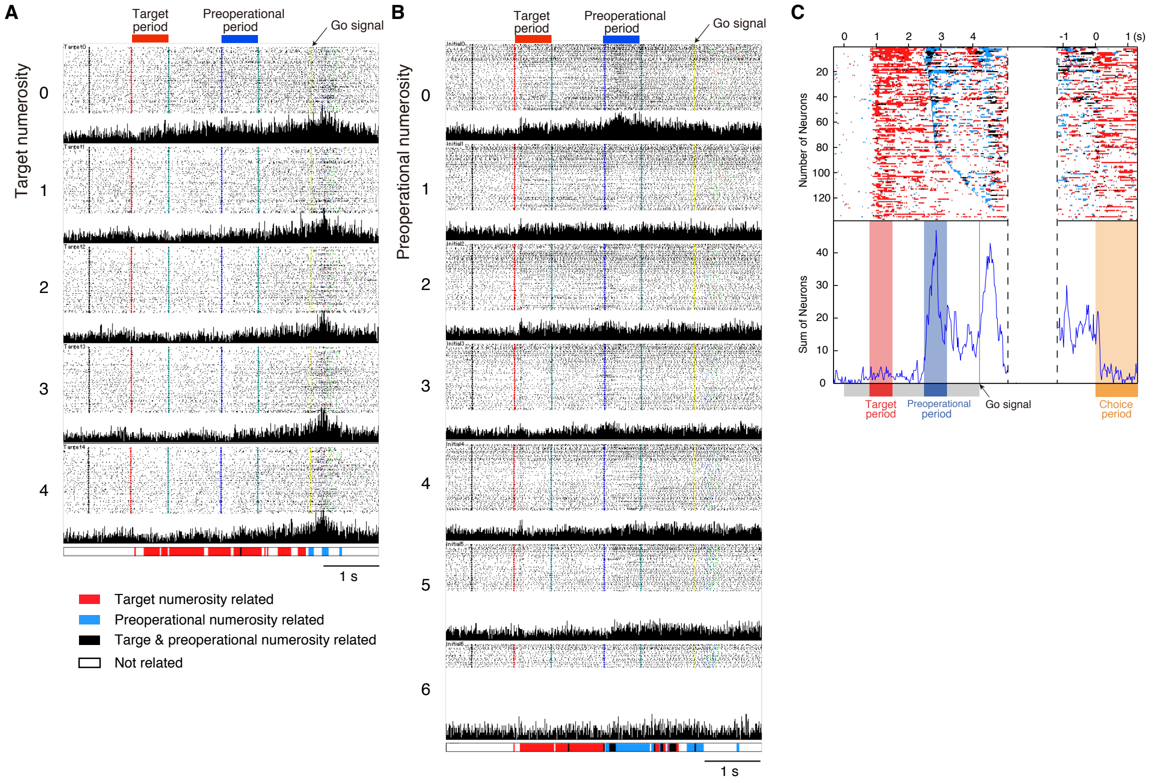

Figure S3 I Example VIP single neurons showing increased discharge for numerosity zero (**A** and **B**). Raster displays and peri-event histograms illustrating the cellular activity for numerosity 0-4 during the target period (red square) and the preoperational period (blue square). Bottom: colors indicate the dynamics of selectivity to each numerosity zero. (**A**) Sustained activity selective to the target numerosity zero from the presentation of the target numerosity to the Delay 2 period. (**B**) Activity selective to the target numerosity zero during the presentation of target numerosity changed selectivity to the preoperational numerosity zero after the presentation of the preoperational numerosity. (**C**) Temporal patterns of the target and the preoperational numerosity zero selectivity (as determined by a sliding three-way ANOVA) as 137 individual zero related neurons (top) and number of preoperational numerosity zero related neurons (bottom). Left: aligned by the onset of fixation period. Right: aligned by the onset of chosen numerosity. Colors are as in A and B.
